# Tumor Endothelial Marker 8 Promotes Proliferation and Metastasis *via* the Wnt/β-Catenin Signaling Pathway in Lung Adenocarcinoma

**DOI:** 10.3389/fonc.2021.712371

**Published:** 2021-10-14

**Authors:** Chen Ding, Jun Liu, Jiali Zhang, Yang Wan, Linhui Hu, Alice Charwudzi, Heqin Zhan, Ye Meng, Huimin Zheng, HuiPing Wang, Youliang Wang, Lihua Gao, Xianwen Hu, Jingrong Li, Shudao Xiong

**Affiliations:** ^1^ Department of Hematology/Oncology Lab, The Second Hospital of Anhui Medical University, Hefei, China; ^2^ Department of Pathology, School of Basic Medical Sciences, Anhui Medical University, Hefei, China; ^3^ Laboratory of Cell Engineering, Beijing Institute of Biotechnology, Beijing, China; ^4^ Department of Emergency, The Second Hospital of Anhui Medical University, Hefei, China; ^5^ Center of Hematology Research, Anhui Medical University, Hefei, China

**Keywords:** TEM8, ANTXR1, LUAD, proliferation, prognosis

## Abstract

Tumor endothelial marker 8 (TEM8), also known as ANTXR1, was highly expressed in cancers, and was identified as a biomarker for early diagnosis and prognosis in some cancers. However, the clinical role and molecular mechanisms of TEM8 in lung adenocarcinoma (LUAD) are still unclear. The present study aimed to explore its clinical value and the molecular mechanisms of TEM8 underlying the progression of LUAD. Our study found the elevation of TEM8 in LUAD cell lines and tissues. What’s more, we observed that the TEM8 expression level was associated with tumor size, primary tumor, and AJCC stage, and LUAD patients with high TEM8 expression usually have a poor prognosis. Then, we conducted a series of experiments by the strategy of loss-of-function and gain-of-function, and our results suggested that the knockdown of TEM8 suppressed proliferation, migration, and invasion and induced apoptosis in LUAD whereas overexpression of TEM8 had the opposite effect. Molecular mechanistic investigation showed that TEM8 exerted its promoting effects mainly through activating the Wnt/β-catenin signaling pathway. In short, our findings suggested that TEM8 played a crucial role in the progression of LUAD by activating the Wnt/β-catenin signaling pathway and could serve as a potential therapeutic target for LUAD.

## Introduction

Lung cancer is the major cause of tumor-related death worldwide (1). Non‐small cell lung cancer (NSCLC), which includes adenocarcinoma, squamous cell carcinoma, and large cell carcinoma, represents approximately 85% of all lung cancer cases (2, 3). Among them, the incidence of lung adenocarcinoma (LUAD) is rising and is gradually occupying a center stage (4). In addition, although molecular targeted therapy and immunotherapy for LUAD have made great progress in recent years, the 5-year overall survival (OS) rate historically remains very poor (5–8). Hence, a deeper understanding of the molecular mechanisms underlying the development of LUAD might establish effective therapeutic targets that are urgently needed.

Tumor endothelial marker 8 (TEM8), an integrin-like cell surface protein, was demonstrated as a tumor-associated marker in colorectal cancer by St. Croix in 2000 (9). Initially, TEM8 was found as an anthrax toxin receptor, so it was alternatively named ANTXR1 (10). A previous study also reported TEM8 as a specific protein molecule upregulated in tumor endothelial cells, required for tumor angiogenesis (11). With the deepening of research, an increasing number of mechanisms of TEM8 in cancer were revealed, such as in gastric cancer (12), breast cancer (13), and ovarian cancer (14). Among these, Tiara et al. (15) investigated the role of TEM8 in cancer progression and metastasis in invasive breast cancer; they proved that TEM8 regulates cancer progression by affecting the expression levels of cell cycle-related genes. Furthermore, in early 2020, researchers had reported that TEM8 might be used as an early diagnostic indicator of lung cancer, providing a reference for the early diagnosis of lung cancer in future clinical practice (16). However, the mechanism and clinical value in LUAD are not clear and need to be elucidated.

In this study, we explored the role and mechanism of TEM8 in LUAD. Our experiments *in vitro* and *in vivo* elucidated that the TEM8 in LUAD cell lines remarkably induced cell proliferation, invasion and migration, and suppressed apoptosis. In addition, our mechanistic investigations showed that TEM8 promoted lung cancer cell proliferation and invasion by activating Wnt/β-catenin. Moreover, we also found that TEM8 was associated with reduced overall survival (OS). Collectively, our results revealed that TEM8 played a crucial role in the progression of LUAD by activating the Wnt/β-catenin signaling pathway and might be a novel biomarker and therapeutic target for LUAD.

## Methods

### Cell Culture and Regents

Human LUAD cell lines (A549, and H1299) and human normal bronchial epithelial cell line (BEAS-2B) were acquired from American Type Culture Collection (Manassas, VA, USA) and maintained in RPMI-1640 medium (Hyclone Logan, Utah, USA) supplemented with 10% fetal bovine serum. All cell lines were maintained at 37°C and 5% CO2 in a humid environment.

ICG001 (SF6827) was purchased from Beyotime Institute of Biotechnology and dissolved in DMSO. And the TEM8-overexpressed cells were treated with ICG001 for 24 hours at the recommended concentration of 25 μM.

### Cell Transfection

SiRNA for TEM8 was designed and synthesized by Gene Pharma (Shanghai, China),and the sequence was: si-TEM8 (1) F: 5’-GCCAGUGAGCAGAUUUAUUTT-3’ (forward), R: 5’-AAUAAAUCUGCUCACUGGCTT-3’ (reverse). si-TEM8 (2) F: 5’-GCGGAUUUGACCUGUACUUTT-3’ (forward), R: 5’-AAGUACAGGUCAAAUCCGCTT-3’ (reverse). The corresponding negative control (NC) sequence was also purchased from the same company: F 5’-UUCUCCGAACGUGUCACGUTT-3’ (forward), R: 5’-ACGUGACACGUUCGGAGAATT-3’ (reverse). The cells were transfected with the siRNA using Lipofectamine 2000 (Life Technologies, Grand Island, NY, USA) according to the manufacturer’s protocol.

Meanwhile, the stably TEM8 overexpressing cell lines and control cell lines were established by lentiviral transfection. Overexpression plasmids lentiviral vector carrying GFP was synthesized by GeneChem (shanghai, China). And the cells were transfected with overexpression plasmids and empty vectors according to the manufacturer’s instructions. Then puromycin (1ug/ml) (Bioss, Beijing, China) was used to select the stably transfected cell lines.

The knockdown and overexpression efficiencies were evaluated by quantitative real-time PCR (qRT-PCR) and western blotting.

### Real-Time Quantitative PCR Analysis

Total RNA was extracted using Trizol reagent (Invitrogen, Carlsbad, CA, USA) an extracted following the manufacturer’s protocol. The cDNA was obtained *via* reverse transcription using the reverse transcription kit (Thermofisher Scientific, USA) according to the manufacturer’s manual. Quantitative real-time PCR (qRT-PCR) was performed using TB Green PCR Mix (TaKaRa, Dalian, China) according to the manufacturer’s manual. The primer sequences are: (1) TEM8: F: 5’-TGCAACACAGAAATGCTCTGCCTG-3’ (forward), R: 5’-TTTATCCCTGGGTGATGAAGCCCA-3’ (reverse), (2) GAPDH: F: 5’-AGCAAGAGCACAAGAGGAAG-3’ (forward), R: 5’-GGTTGAGCACAGGGTACTTT-3’ (reverse). The relative expression ratio of TEM8 in each group was calculated by the 2^-ΔΔCt^ method.

### Western Blot

The cells were collected, and total proteins were extracted. The total proteins were resolved by SDS-polyacrylamide gel electrophoresis on 12% gels and transferred onto polyvinylidene difluoride transfer membrane (Merck Millipore, Billerica, USA). Primary antibody incubation was performed overnight at 4°C. The primary antibodies used were TEM8 (1:500 dilution) (Affinity, America), Wnt1 (1:1000 dilution) (Abcam, Cambridge, UK), β-catenin, pGSK-3β, GSK-3β, cyclin D1, P21, β-actin (1:1000 dilution) (Cell Signaling Technology, Danvers, MA, USA). Then, the blots were developed with a peroxidase-conjugated secondary antibody (1:5000 dilution) (Cell Signaling Technology, Danvers, MA, USA), and the proteins were visualized using the ECL Plus detection Reagent (Tanon, Shanghai, China). The gray-scale value was assessed by *ImageJ* (ImageJ v1.47).

### Flow Cytometry Analysis

Flow cytometry analysis was conducted to detect apoptotic cells. After transfecting the lung cancer cell lines with TEM8-siRNA and NC-siRNA, the cells were harvested into centrifuge tubes. The cells were then washed with PBS and stained using 5ul Annexin V reagents and 5ul PI reagents (BestBio, Shanghai, China) for 15min in the light-proof condition. The mixture was added to 400ul of Binding Buffer after the reaction. Cell apoptosis was analyzed using the *CytoFlex* (Beckman CytoFlex, USA).

### Cell Proliferation Assay

The cell counting kit-8 (CCK-8) assay was used to evaluate the level of cell proliferation. After 24h of transfection with TEM8 siRNA and NC siRNA, the cells were cultured in 96-well plates (5×10^4^/well). The cells were then incubated for 24, 48, and 72h at 37°C. Thereafter, 10µl of CCK−8 solution (Beyotime Institute of Biotechnology, China) was added to each well and cultured at 37°C for 2h according to the manufacturer’s instruction. The absorbance at 450nm was measured using a microplate autoreader (Bio Tek Instruments Inc., Winooski, VT, USA).

### Colony Formation Assay

For the colony formation assays, the transfected cells (1×10^3^/well) were placed into six-well plates and cultured for two weeks. The colonies were then washed with PBS, fixed in methanol, stained with crystal violet, photographed, and counted.

### Wound-Healing Assay

Cells (1×10^5^/well) were pretreated or not with mitomycin C (5ug/ml) (Selleck, USA), seeded in six-well plates, and serum-starved for 24h. Then we used a 200ul pipette tip to make a scratch after cells were grown to 80% confluence. The wound healing process was observed and photographed at a magnification of 100× at the indicated time points (0 and 24h).

### Transwell Invasion Assay

Cells (10×10^4^/well) were pretreated with mitomycin C(5ug/ml) (Selleck, USA), and seeded in the top chambers in 100μL serum-free medium; the lower chambers were filled with a 600ul complete medium with 20% FBS. After 48h incubation, 0.1% crystal violet dye was used to stain cells. The images were analyzed by *ImageJ* (ImageJ v1.47).

### Dual-Luciferase Report Assay

Cells (5×10^3^/well) were seeded into a 96-well plate. And the cells of the knockdown group were transfected with 200ng Top/Fop-flash reporter plasmids (Beyotime Institute of Biotechnology, China) and 0.25ul siRNA using Lipofectamine 2000. The stably selected overexpression cells were just transfected with 200ng Top/Fop-flash reporter plasmids using Lipofectamine 2000. After 24h of incubation, the luciferase activity was detected by the Dual-Luciferase Reporter Assay System (Beyotime Institute of Biotechnology, China).

### Immunohistochemistry

IHC staining was performed as described previously (17). The Human LUAD tissue array was purchased from Outdo Biotech (Shanghai, China). TEM8 expression was assessed by multiplying scores representing the reaction degree of positive cells and staining intensity. Staining intensity was graded as 0 (no staining), 1 (weak staining=light yellow), 2 (moderate staining=yellow brown), and 3 (strong staining=brown). The extent (0–100%) of reactivity was scored as follows: 0 (≤25% positive cells), 1 (26%-50% positive cells), 2 (51%-74% positive cells), 3 (≥75% positive cells). Scores of 0–4 were classified as low expression, whereas all other scores were classified as high expression. Two pathologists without knowledge of the clinic-pathological variables independently scored staining on each slide. Staining assessment and the allocation of tumors by the two pathologists were similar. All immunohistochemical images were obtained using an Olympus BX51 microscope equipped with a 200× objective lens (Olympus) and a DP 50 camera (Olympus).

### *In Vivo* Tumorigenic Assay

Female BALB/c nude mice (4 weeks old) were purchased from GemPharmatech Co.Ltd (Nanjing, China) and maintained under specific pathogen-free conditions. The mice were randomly assigned into NC and TEM8-knockdown groups (n=5 mice/group). H1299 cells were transfected with siRNA-TEM8 or siRNA-NC for 24h. And cells were collected with PBS and mixed with an equal volume of Matrigel at a final concentration of 1×10^8^/ml. Then each mouse was injected with 100ul of the cell suspensions. When the volumes of tumors grew to about 1 cm^3^, the nude mice were killed, and the tumor was extracted. Meanwhile, the tumor volumes and body weights were measured every 3 days. All animal studies were carried out with the approval of the Ethics Committee of AnHui Medical University (Approval No. LLSC20190462).

### Statistical Analysis

Statistical analyses were performed using SPSS (version 16.0 for Windows, SPSS Inc., Chicago, IL, USA) and GraphPad Prism 8.0 (GraphPad 8.0, San Diego, CA, USA). The quantitative data were expressed as means ± SD. Significant differences were determined by the independent t-test or ANOVA. Survival curves were estimated by the Kaplan–Meier (KM) method. Chi-square analysis was used to explore relations between TEM8 expression in tumor tissues and clinicopathologic characteristics of LUAD patients. The independent prognostic factors in LUAD were determined by Cox regression. And factors with P<0.05 in the univariate analysis were entered into the multivariate analysis. Regarding Kaplan-Meier plotter (http://kmplot.com/) analysis, patients with adenocarcinoma were chosen; the patients were divided into 2 groups based on the median value of TEM8, higher than median TEM8 value was defined as a high group, otherwise was assigned a low group. What’s more, the gene expression data set was derived from 220092_s_at probe. A p-value less than 0.05 was used as the criterion for statistical significance.

## Results

### TEM8 Was Upregulated in LUAD Tissues and Cell Lines

**Figure 1:**
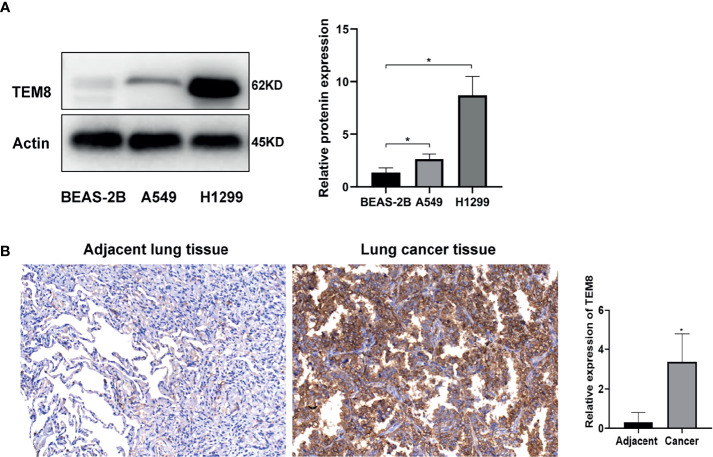
TEM8 expression is increased in LUAD tissue and LUAD cell lines. **(A)** Relative expression of TEM8 protein in human LUAD cell lines (A549 and H1299) and normal bronchial epithelial cell lines (BEAS-2B) by western blotting. **(B)** Representative images and quantitative results of TEM8 expression in LUAD tissue and adjacent normal tissue by immunohistochemistry. All experiments were repeated at least three times and representative as shown. Data are means ± SD, *p < 0.05.

To determine whether TEM8 is aberrantly expressed, we performed Western Blot by using A549, H1299 and BEAS-2B cells. Our data showed that TEM8 was highly expressed in LUAD cell lines (A549 and H1299) compared with normal bronchial epithelial cell lines (BEAS-2B) (**Figure 1A**). To investigate the level of TEM8 expression in patients with LUAD, we applied IHC to examine its expression in a human LUAD tissue array (HLugA180Su07). We found TEM8 remarkably upregulated in the tumor tissues compared with the matched adjacent normal tissues (**Figure 1B**). Taken together, these findings suggested that TEM8 was significantly elevated in LUAD tissues and cell lines.

### High TEM8 Expression Was Associated With Poor Prognosis of LUAD Patients

**Figure 2:**
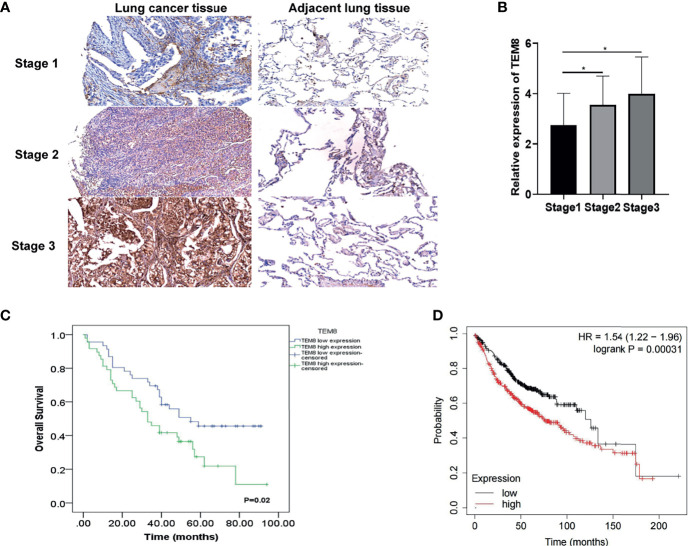
TEM8 expression correlated with poor prognosis in LUAD patients. **(A)** Representative images of TEM8 IHC staining in samples from human LUAD tissue array (magnification, 200×). **(B)** Quantitative results of TEM8 expression levels in LUAD tissue arrays. **(C, D)** Kaplan-Meier curves of overall survival in LUAD patients stratified by TEM8 expression. Each subgroup was divided into low and high TEM8 expression groups. Patients with higher TEM8 expression had a poorer prognosis. All experiments were repeated at least three times and representative as shown. Data are means ± SD, *p < 0.05.

Based on the above analysis, we further investigated whether TEM8 expression is associated with the clinical outcome of LUAD. As presented in **Figures 2A, B**, the expression levels of TEM8 correlated with the tumor stage. Given this, we deeply analyzed the human LUAD tissue array (HLugA180Su07), which contained gene expression profiles of 95 human LUAD with clinical follow-up information. The samples were divided into two groups (low and high) according to the IHC staining score. As shown in the KM survival analysis, LUAD patients with high-TEM8 expression usually had shorter OS (**Figure 2C**). The result of the Kaplan-Meier plotter (http://kmplot.com/) confirmed our finding, demonstrating that high TEM8 expression remarkably correlated with a poorer OS in patients with LUAD (HR= 1.54; 95% CI, 1.22-1.96; P = 0.00031; **Figure 2D**).

Notably, TEM8 expression was correlated with tumor size (p<0.05), primary tumor (p<0.05), and AJCC stage (p<0.05) based on the Chi-square test (**Table 1**). As presented in the univariate Cox regression analysis, TEM8 expression, primary tumor, and AJCC stage were risk factors for LUAD (P < 0.05). But the multivariate Cox regression analysis indicated that TEM8 expression was not an independent predictor of OS (**Table 2**). Anyway, all these results proved that TEM8 expression was upregulated in human LUAD tissues, which may be associated with poor prognosis in LUAD.

**Table 1:** Correlation between TEM8 expression in tumor tissues and clinicopathologic characteristics of LUAD patients.

Characteristic	Patients	TEM8 expression		P-value
		Low (n = 46)	High (n = 49)	
Gender				0.784
Male	53	25	28	
Female	42	21	21	
Age (years)				0.363
<60	45	24	21	
≥60	50	22	28	
Tumor size (cm)				0.000*
<4	43	36	8	
≥4	51	10	41	
Primary tumor (T)				0.000*
T_1_ – T_2_	67	44	23	
T_3_ – T_4_	26	0	26	
Tumor stage				0.199
I+II	62	33	29	
III+IV	33	13	20	
AJCC stage				0.001*
1-2 stage	50	32	18	
3-4 stage	44	13	31	

*represent P value less than 0.05.

**Table 2:** Univariate and multivariate analyses of prognostic factors in LUAD patients.

Characteristic	Univariate analysis			Multivariate analysis		
	HR	95% CI	P-value	HR	95% CI	P-value
Gender						
Male *vs.* Female	1.110	0.668-1.844	0.688			
Age (years)						
<60 *vs.* ≥60	1.451	0.868-2.426	0.156			
Tumor size (cm)						
<4 *vs.* ≥4	1.229	0.839-1.800	0.289			
Primary tumor (T)						
T1–T2 *vs.* T3–T4	1.875	1.089-3.229	0.023*	1.196	0.659-2.170	0.555
Tumor stage						
I+II *vs.* III+IV	1.016	0.598-1.725	0.953			
AJCC stage						
1-2 *vs.* 3-4	3.493	2.029-6.013	0.000*	3.736	1.773-7.871	0.001*
TEM8 expression						
Low *vs.* High	1.239	1.011-1.518	0.039*	1.132	0.837-1.532	0.422

*represent P value less than 0.05.

### TEM8 Promoted Malignancy Phenotypes of Lung Cancer Cells *In Vitro*

**Figure 3:**
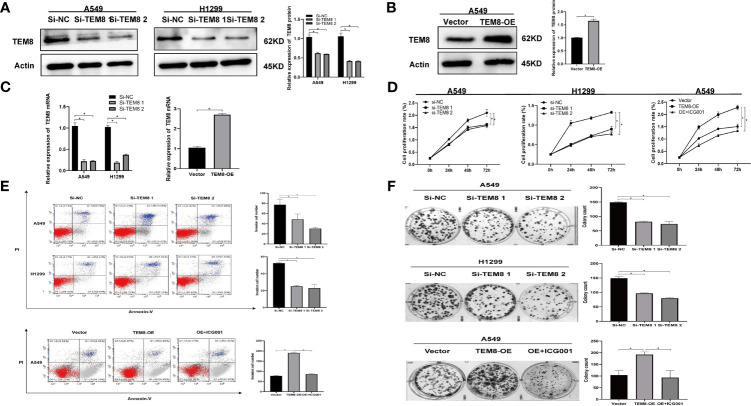
Effects of expression of TEM8 on LUAD cell proliferation and apoptosis *in vitro*. **(A–C)** Knockdown of TEM8 in H1299 and A549 cells and overexpression of TEM8 in A549 cells were identified by quantitative PCR and western blot. **(D, F)** CCK8 and colony formation assays were carried out in A549 and H1299 cells expressing the negative control or siRNA of TEM8 and in A549 cells expressing the vector control, TEM8-OE or OE+inhibitor ICG0001. **(E)** Flow cytometry assays were used to examine the effect of TEM8 on cell apoptosis. All experiments were repeated at least three times and representative as shown. Data are means ± SD, *p < 0.05.

Next, to explore the impact of TEM8 on the malignancy development of LUAD, we constructed the loss-of-function in both H1299 and A549 cells and gain-of-function in A549 cells. After TEM8 silencing with siRNA (**Figures 3A, C**), TEM8 was expressed at a lower level in Si-TEM8 groups than in the NC group. The results of CCK-8 assays showed that the proliferation rates of repressed TEM8 cells presented a remarkable decrease relative to the NC cells (**Figure 3D**). The data revealed that suppressing TEM8 levels reduced the number of proliferative LUAD cells. Moreover, clone formation assay proved that the colony formation abilities also decreased after knocking down TEM8 (**Figure 3E**). In addition, apoptosis assay was performed by the flow cytometry analyses, and our data showed that the apoptotic rate of LUAD cells in the si-TEM8 group was higher than that in the NC group (**Figure 3F**).

Meanwhile, we performed overexpression experiments proved by western blotting and qRT-PCR (**Figures 3B, C**). As expected, the CCK8 and colony formation assays suggested that the ability of LUAD cell proliferation was enhanced as the overexpression level of TEM8 (**Figures 3D, F**). What’s more, the results of the apoptosis assay were opposite to the results obtained when the TEM8 was knocked down (**Figure 3E**).

Together, these results collectively proved that TEM8 accelerates the LUAD cells’ proliferation and decreases apoptosis.

### TEM8 Accelerated the Metastatic Potentials of LUAD Cells

**Figure 4:**
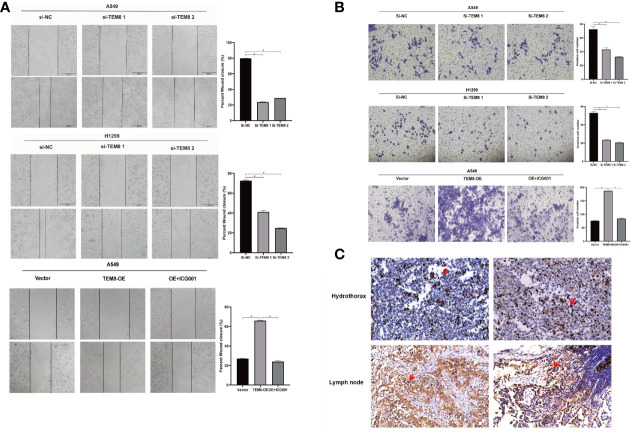
The inhibition of TEM8 suppresses the metastatic potentials of LUAD. **(A, B)** Representative images of cell migration based on wound-healing assays and transwell assays in knockdown groups and overexpression groups, and quantitative analysis of cell migration based on transwell assays. **(C)** Representative images of TEM8 IHC staining in samples from patients with hydrothorax and lymph node metastasis (magnification, 200×). All experiments were repeated at least three times and representative as shown. Data are means ± SD, *p < 0.05.

As mentioned above, the elevated expression level of TEM8 might contribute to the development of LUAD. We next investigated the effect of TEM8 on LUAD metastasis. Firstly, we treated cells with mitomycin C to exclude the effects of proliferation on metastasis, and the wound-healing assays showed that TEM8 deficiency reduced the wound closures of LUAD cells when compared with NC cells, whereas the TEM8-overexpressed A549 cells exhibited stronger migration capabilities than control vector cells (**Figure 4A**). Moreover, we conducted transwell analyses, and the results also certified that the suppression of TEM8 resulted in markedly decreased invasive cell numbers. In contrast, cells stably overexpressing TEM8 showed stronger invasion ability (**Figure 4B**). Above all, these findings proved that TEM8 had a facilitation effect on LUAD cell migration and invasion.

Besides, we performed IHC for TEM8 on 20 pleural fluid samples and 20 lymph node metastasis samples obtained from patients with LUAD. We found that TEM8 was highly expressed in cancer cells in the hydrothorax and lymph node metastasis specimens from late-stage LUAD patients (**Figure 4C**). This result further suggested that TEM8 induced the metastatic potentials of LUAD.

### TEM8 May Promote LUAD Progression Through The Wnt/β-Catenin Signaling Pathway

**Figure 5:**
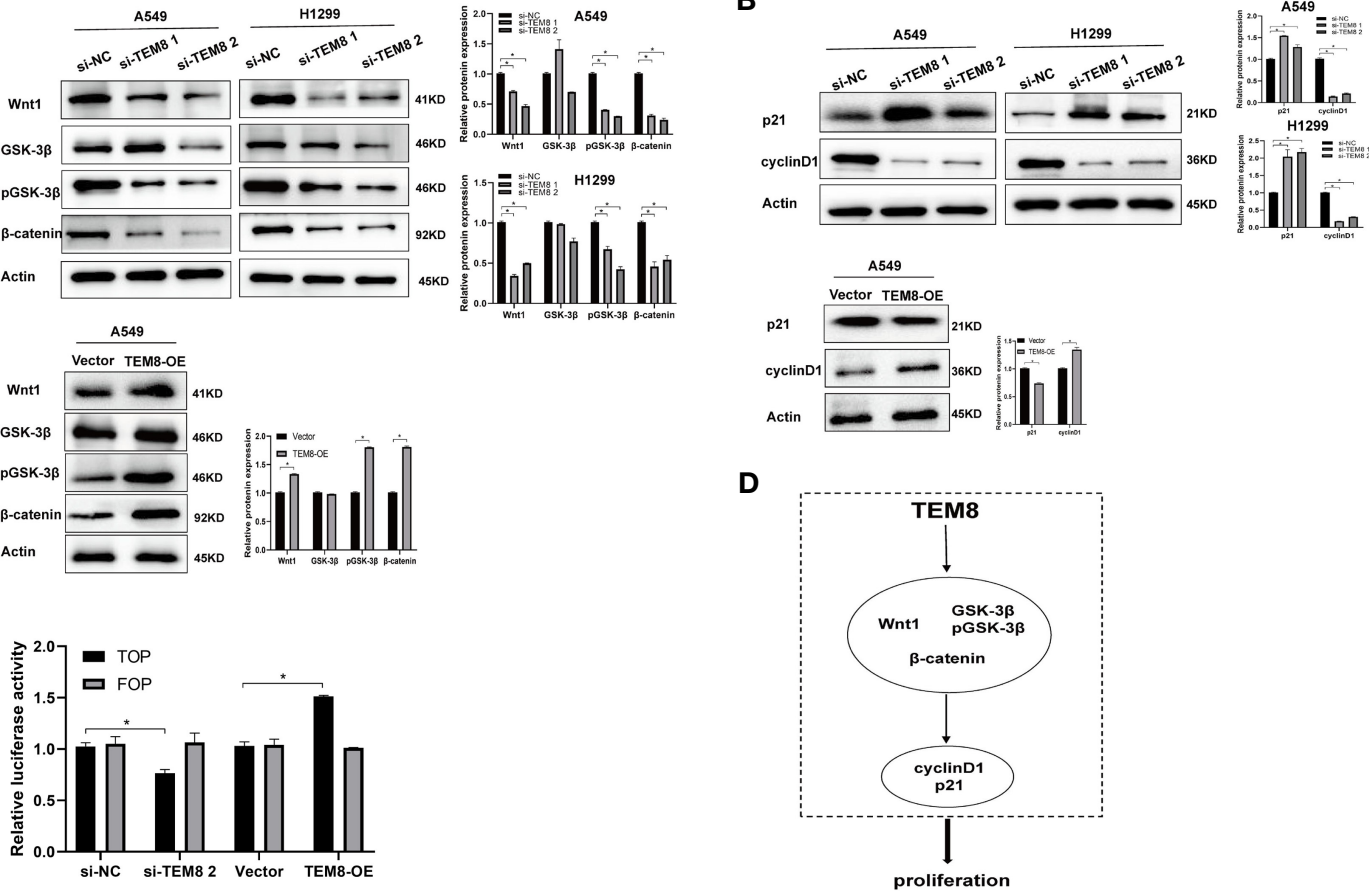
TEM8 may promote LUAD progression *via* activating the Wnt/β-catenin signaling pathway. **(A)** Western blotting was used to investigate the effect of TEM8 downregulation or overexpression on the expression levels of Wnt1, GSK-3β, pGSK-3β and β-catenin in A549 and H1299 cells. **(B)** Western blot analysis for p21 and cyclinD1 in TEM8-silenced cells, TEM8-overexpressed cells, and TEM8-overexpressed cells treated with ICG001. **(C)** The luciferase activity of Wnt/β-catenin signaling was detected using a TOP-flash assay with FOP-flash as a negative control. **(D)** Schematic diagram of the regulatory mechanism of TEM8 in promoting lung cancer cells proliferation. TEM8 could activate the Wnt/β-catenin signaling pathway, then activate the p21 and cyclin D1, thereby mediating the proliferation of LUAD cells. All experiments were repeated at least three times and representative as shown. Data are means ± SD, *p < 0.05.

After elucidating the effect of TEM8 on LUAD progression, we investigated the mechanisms involved in this process. Some reports show the constant upregulation of the Wnt/β-catenin signaling pathway in various cancers (18). Evidence suggests that the Wnt/β-catenin signaling pathway is involved in the proliferation and apoptosis of lung cancer (19). Therefore, we further explored if the tumorigenic effects of TEM8 were dependent on the Wnt/β-catenin signaling pathway. As shown in **Figure 5A**, the expression levels of Wnt1, GSK3β, pGSK3β, and β-catenin were decreased in the TEM8-knockdown (KD) groups but increased in TEM8-overexpressed groups. To further confirm these results, rescue experiments were performed. We used the Wnt/β-catenin signaling pathway inhibitor ICG001 to treat TEM8-overexpressed A549 cells. Then we found that ICG001 significantly reduced proliferation capacity in the CCK8, colony formation, and apoptosis assays (**Figures 3D–F**). Also, ICG001 treatment inhibited the promotional effects on migration and invasion caused by TEM8 overexpression (**Figures 4A, B**). Importantly, we also used the dual-luciferase reporter assay to investigate the regulation of Wnt/β-catenin signaling activity. As shown in **Figure 5C**, the results showed that TEM8 knockdown significantly reduced Wnt reporter activity. In contrast, the overexpression of TEM8 enhanced Wnt reporter activity. Collectively, these results consistently proved that TEM8 played a key role in the activation of the Wnt/β-catenin signaling pathway.

In addition, previous studies have proved that p21 and cyclin-D1 are the Wnt targets associated with the proliferation of tumor cells (20–22). Therefore, we investigated whether TEM8 activated the Wnt/β-catenin signaling pathway to regulate the p21 and cyclin D1. Expectedly, the results proved that the expression level of p21 in the Si-TEM8 groups was higher compared with the Si-NC groups. In contrast, the expression of p21 was remarkably downregulated in the TEM8-overexpressed group. Additionally, the cyclin D1 expression level was lower with TEM8 knockdown but higher with TEM8 overexpression (**Figure 5B**). Above all, our findings indicated that the Wnt/β-catenin pathway might be the major downstream signaling pathway activated by TEM8 (**Figure 5D**).

### TEM8 Promotes LUAD Cell Tumorigenesis *In Vivo*

**Figure 6:**
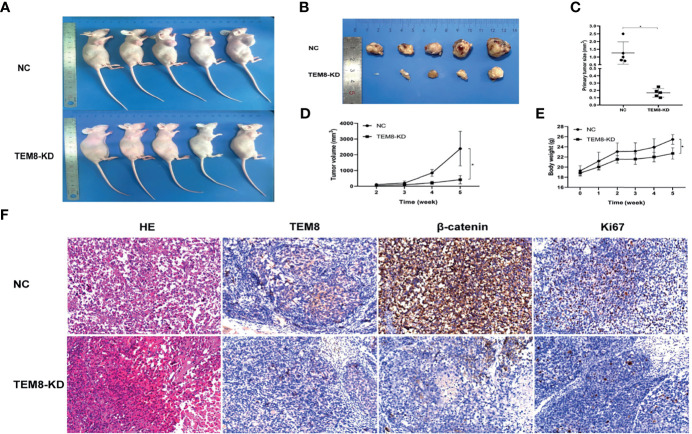
TEM8 promotes tumorigenesis of LUAD cells in nude mice. **(A, B)** Knockdown (KD) of TEM8 attenuated tumor growth in the nude mice model by xenograft growth assay. Xenograft tumors **(B)** from **(A)** were dissected and photographed. **(C)** Tumor size from the negative control or TEM8-knockdown groups. Each group contained five mice. **(D, E)** The tumor growth curve and mice growth curve of TEM8-knockdown cells were compared with negative control cells. Each group contained five mice. **(F)** HE staining and immunohistochemistry for TEM8, β-catenin, and Ki67 in the negative control group and the TEM8-knockdown group. Data are means ± SD, *p < 0.05.

To investigate the effect of TEM8 on LUAD cell tumorigenesis *in vivo*, we injected mice with H1299 cells expressing si-NC and si-TEM8 *via* subcutaneous injections. As shown in **Figures 6A, B**, the H1299/TEM8-KD-injected animals had fewer and smaller tumors than the H1299/NC-injected animals. Additionally, as indicated by the xenograft tumor size, tumor growth curves, and mice body weight curves (**Figures 6C–E**), TEM8-KD cells had a significantly weaker capacity to form tumor nodules in nude mice. The findings suggest TEM8 expression is critical for the development of LUAD cells. The tissues were further examined by HE staining to confirm the presence of tumors. IHC demonstrated that TEM8-KD groups had fewer expression levels of TEM8 than NC groups. Moreover, to verify if TEM8 exerts its effect by activating Wnt/β-catenin signaling pathway *in vivo*, the expression of β-catenin was examined in the xenograft tumor samples, and the results showed that β-catenin was expressed in the cell nucleus at a lower level in the TEM8-KD group. Additionally, the IHC also found that the TEM8-KD group had fewer Ki67-positive cells than the NC group (**Figure 6F**). All these data suggested that TEM8 is capable of promoting the development in LUAD.

## Discussion

TEM8, an anthrax toxin receptor, is reported as a tumor-associated biomarker in cancers (10, 23), however, the role and mechanism in LUAD are not clear. In our study, our results demonstrated the elevation of TEM8 in LUAD cell lines and cancer tissues. Our data also found that the TEM8 expression level was associated with cancer behaviors, including tumor size, primary tumor, and AJCC stage, and TEM8 is a poor prognosis factor in LUAD patients. Importantly, we firstly reported that the TEM8 promoted LUAD proliferation and metastasis *via* the Wnt/β-catenin signaling pathway.

Previous studies have proved that TEM8 is broadly expressed in tumor endothelial cells (9, 11). Meanwhile, some researchers found that TEM8 positively regulates the proliferation of endothelial cells in cancer while negatively regulating the proliferation in normal endothelial cells (24, 25). But few reports indicated that TEM8 is highly expressed in lung cancer (16, 26). In the study of Sun (16), they measured the serum TEM8 expression in 204 patients with lung cancer by PCR, and the results showed that the expression level of TEM8 in patients with lung cancer was significantly higher than that in healthy subjects. Their report was based on serum TEM8 levels, and LUAD was not mentioned, so the results and conclusions need to be elucidated in LUAD tissues. In addition, Gong et al. (26) also revealed that TEM8 was highly expressed in lung cancer tissues and cells and potentially involved in tumor angiogenesis. Thus, our results about the expression level of TEM8 were roughly consistent with the two previous studies and suggested that a high level of TEM8 may induce progression in LUAD.

Our data also found that the TEM8 expression level was associated with cancer behaviors, including tumor size, primary tumor, and AJCC stage in LUAD patients. The univariate Cox regression analysis demonstrated that TEM8 expression, primary tumor, and AJCC stage were risk factors for LUAD. However, TEM8 was not the independent risk factor of OS for LUAD by multivariate Cox regression analysis, this maybe caused by the partly missing clinical data including Eastern Cooperative Oncology Group (ECOG) Scale of performance status in the patients’ tissue array samples, so a larger sample size and longer follow-up time are needed to demonstrate the clinical value in the future. Simultaneously, a recent study has shown that TEM8 could be an excellent indicator for early clinical diagnosis and prognosis of lung cancer (16). In the study, they plotted the receiver operating characteristic (ROC) curve and found that TEM8 has a diagnostic value in patients with lung cancer (pathological type was not mentioned). In the light of their study, TEM8 expression correlated with smoking, lymphatic metastasis, TNM stage, and pleural invasion. Anyway, our findings extended previous observations and supported the notion that TEM8 expression level was associated with cancer behavior and was a poor factor in LUAD.

Recent studies have reported that TEM8 positively regulates the proliferation in varieties of cancers, including breast cancer, gastric cancer, and so on (12, 14, 27–29). Subsequently, our loss-of-function and gain-of-function experiments and a series of functional experiments *in vitro* and *in vivo* indicated that TEM8 accelerates the proliferation but suppresses the apoptosis of LUAD cells. Also, the elevation of TEM8 has a facilitating effect on the migration and invasion of LUAD cells. What’s more, higher TEM8 expression was validated in the pleural effusion and lymph nodes from the late stage of LUAD patients. These results were similar to Gong’s previous study (26). Thus, all these data suggested that TEM8 plays a vital role in the development of LUAD.

Moreover, we further set up experiments to explore the underlying mechanism through which TEM8 promotes LUAD progression. Studies have reported that Wnt/β-catenin signaling is important in LUAD cell lines, and the inhibition of Wnt reduces proliferation (30–33). Thus, we speculated that TEM8 might activate the Wnt/β-catenin signaling pathway to promote the development of LUAD. In this study, we verified the above signaling pathway proteins in the TEM8-knockdown and TEM8-overexpression groups. The results suggested that the levels of Wnt1, pGSK-3β, and β-catenin were decreased in the TEM8-knockdown group but increased in the TEM8-overexpression group. Additionally, Wnt/β-catenin inhibitor ICG001 rescued the function of overexpressed TEM8 in LUAD cells. Importantly, as expected, the data validated that the TEM8 knockdown inhibited the activity of the Wnt/β-catenin signaling pathway *via* the TOP/FOP flash luciferase reporter system. Hence, these findings collectively indicated that TEM8 promoted the malignant biological behavior of LUAD cells by activating the Wnt/β-catenin signaling pathway.

Moreover, previous pieces of evidence have demonstrated that TEM8 promoted the proliferation of osteosarcoma and ovarian cancer by regulating the expression of p21 and cyclin D1 (14, 27). Meanwhile, multiple previous studies have demonstrated that cyclinD1 and p21 are the downstream target genes of the Wnt/β‐catenin signaling pathway (34–38). Besides, we found that the expression level of cyclin D1 was reduced while the expression level of p21 was increased in TEM8-siRNA cells. Furthermore, these results in the TEM8-OE group contradicted those in the TEM8-KD group. Above all, these findings firstly demonstrated that TEM8-induced progression of LUAD may be due to modulation of Wnt/β-catenin activity. Our novel findings may provide new insights into the mechanisms of TEM8 and suggest TEM8 could be a potential therapeutic target of LUAD.

This study aimed to systematically explore the clinical role and the molecular mechanisms of TEM8 underlying the progression of LUAD and establish effective therapeutic targets. However, due to the limited conditions of this study, there are still some shortcomings in the relevant clinical prognostic analysis. Therefore, additional studies are needed to refine and expand our findings.

In conclusion, our study showed that the expression of TEM8 in LUAD was significantly upregulated and closely associated with the poor prognosis of LUAD patients. Simultaneously, our results firstly demonstrated that TEM8 played a crucial role in promoting LUAD cell progression by activating the Wnt/β-catenin signaling pathway *in vitro* and *in vivo*. Our findings suggested that TEM8 could serve as a biomarker and potential therapeutic target for LUAD.

## Data Availability Statement

The original contributions presented in the study are included in the article/**Supplementary Material**. Further inquiries can be directed to the corresponding authors.

## Ethics Statement

The animal study was reviewed and approved by Anhui Medical University.

## Author Contributions

SX and JuL designed the study. CD and JuL performed the experiments. CD and JuL interpreted the results. JZ and YaW supervised the study. SX provided the funding. CD and LH wrote the draft. AC and YM revised the draft. All authors contributed to the article and approved the submitted version.

## Funding

This work was partly supported by the Key Research and Development Plan of Anhui Province, China (201904a07020058), Higher School of Anhui Provincial Natural Science Research Project (KJ2018A0198), Foundation of Anhui Medical University (2019xkj134), National Science Foundation of China (81272259, 82172858), Basic and Clinical Cooperative Research Promotion Plan of Anhui Medical University[2020xkjT021], Scientific Research Foundation of the Institute for Translational Medicine (SRFITMAP, 2017zhyx13).

## Conflict of Interest

The authors declare that the research was conducted in the absence of any commercial or financial relationships that could be construed as a potential conflict of interest.

## Publisher’s Note

All claims expressed in this article are solely those of the authors and do not necessarily represent those of their affiliated organizations, or those of the publisher, the editors and the reviewers. Any product that may be evaluated in this article, or claim that may be made by its manufacturer, is not guaranteed or endorsed by the publisher.

## Supplementary Material

Supplementary Figure 1 The rate of pGSK-3β/GSK-3β was decreased with the knockdown of TEM8 **(A, B)**, whereas elevated with the overexpression of TEM8 **(C)**.

Supplementary Figure 2 Nuclear localization of β-catenin is observed in IHCs of patient samples.

Supplementary Figure 3 The expression of TEM8 in lung cancer cell lines (A549, H1299, H1395, and 95D) and normal bronchial epithelial cell lines.

## Abbreviations

LUAD, Lung adenocarcinoma; NSCLC, Non‐small cell lung cancer; TEM8, Tumor endothelial marker 8; ANTXR1, Anthrax toxin receptor; AJCC, American Joint Committee on Cancer; OS, Overall survival; CI, Confidence Intervals; HR, Hazard Ratio; NC, Negative control; siRNA, Small interfering RNA; qRT-PCR, Quantitative real-time PCR; pGSK-3β, Phosphorylated GSK-3β; Phosphorylated ERK1/2; CCK-8, Cell counting kit-8; PBS, Phosphate buffered solution; FBS, Fetal bovine serum; IHC, Immunohistochemistry; KM, Kaplan–Meier; ROC, Receiver operating characteristic; ECOG, Eastern Cooperative Oncology Group.
