# Supplementary figures and images for: Tumor Endothelial Marker 8 Promotes Proliferation and Metastasis via the Wnt/β-Catenin Signaling Pathway in Lung Adenocarcinoma
Source: Front Oncol. 2021 Oct 14;11:712371. doi: 10.3389/fonc.2021.712371 (PMC8552045; doi:10.3389/fonc.2021.712371)

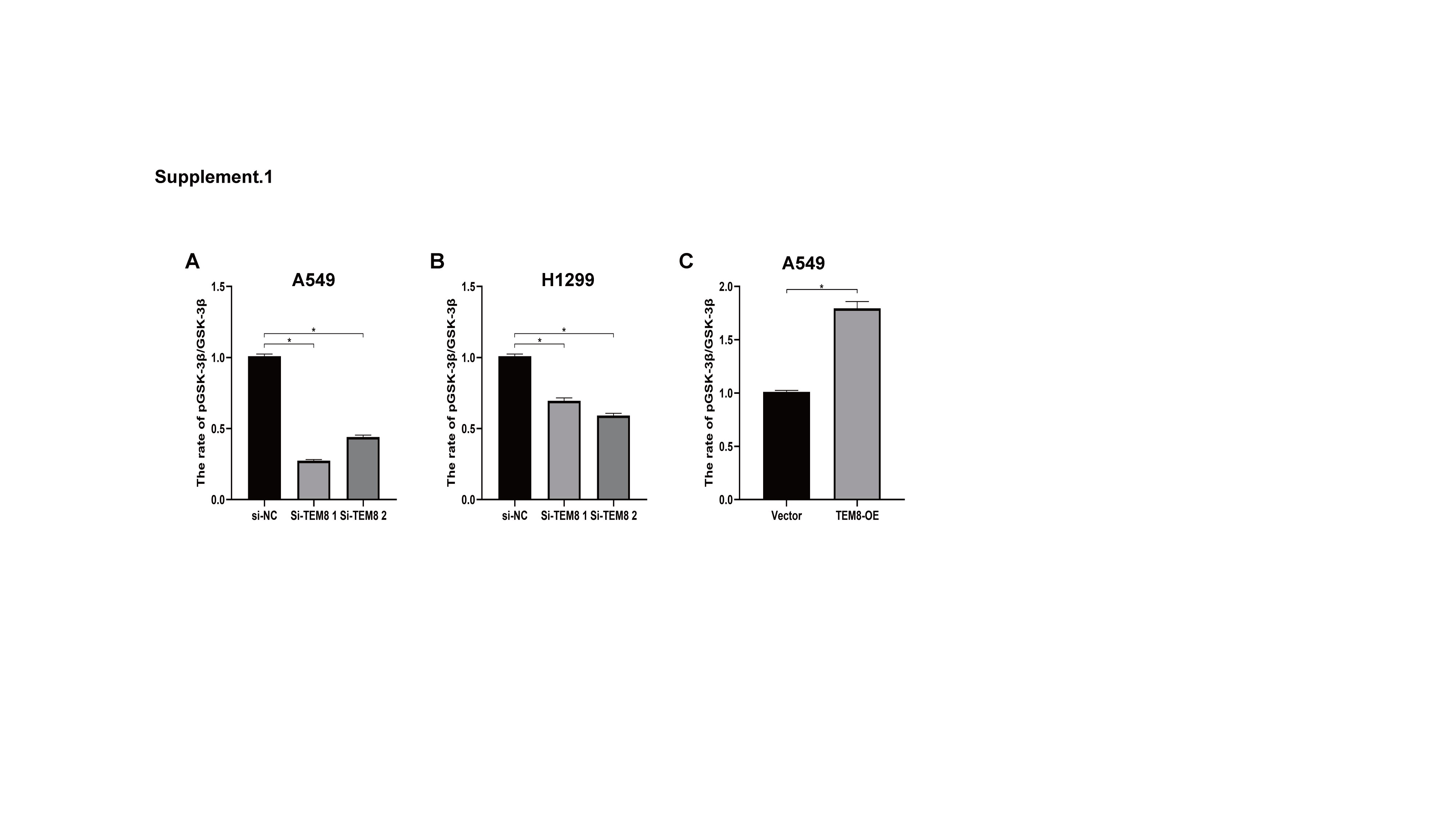

Supplement: Supplementary Figure 1 — The rate of pGSK-3β/GSK-3β was decreased with the knockdown of TEM8 (A, B), whereas elevated with the overexpression of TEM8 (C). [file Image_1.tif]

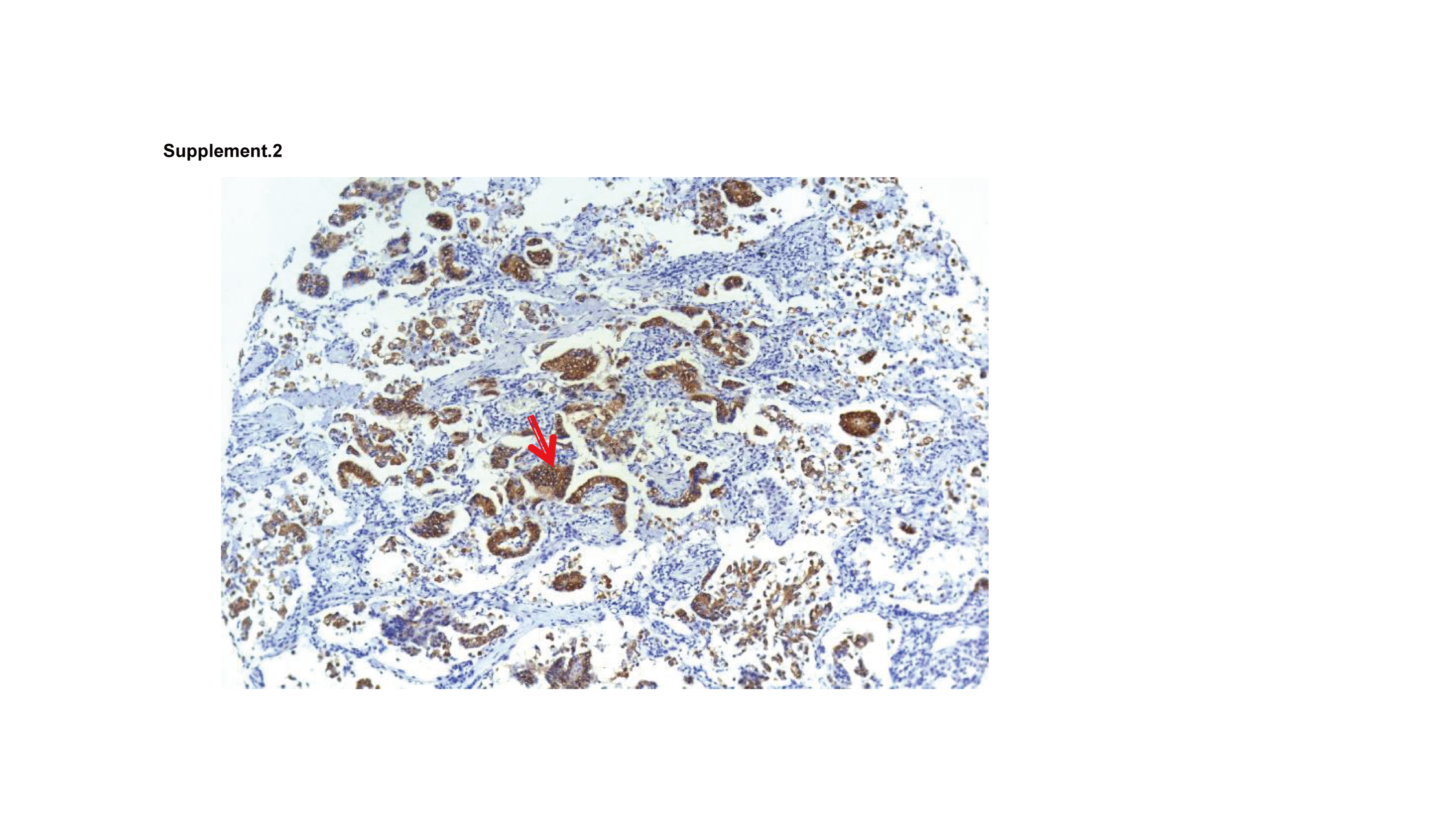

Supplement: Supplementary Figure 2 — Nuclear localization of β-catenin is observed in IHCs of patient samples. [file Image_2.tif]

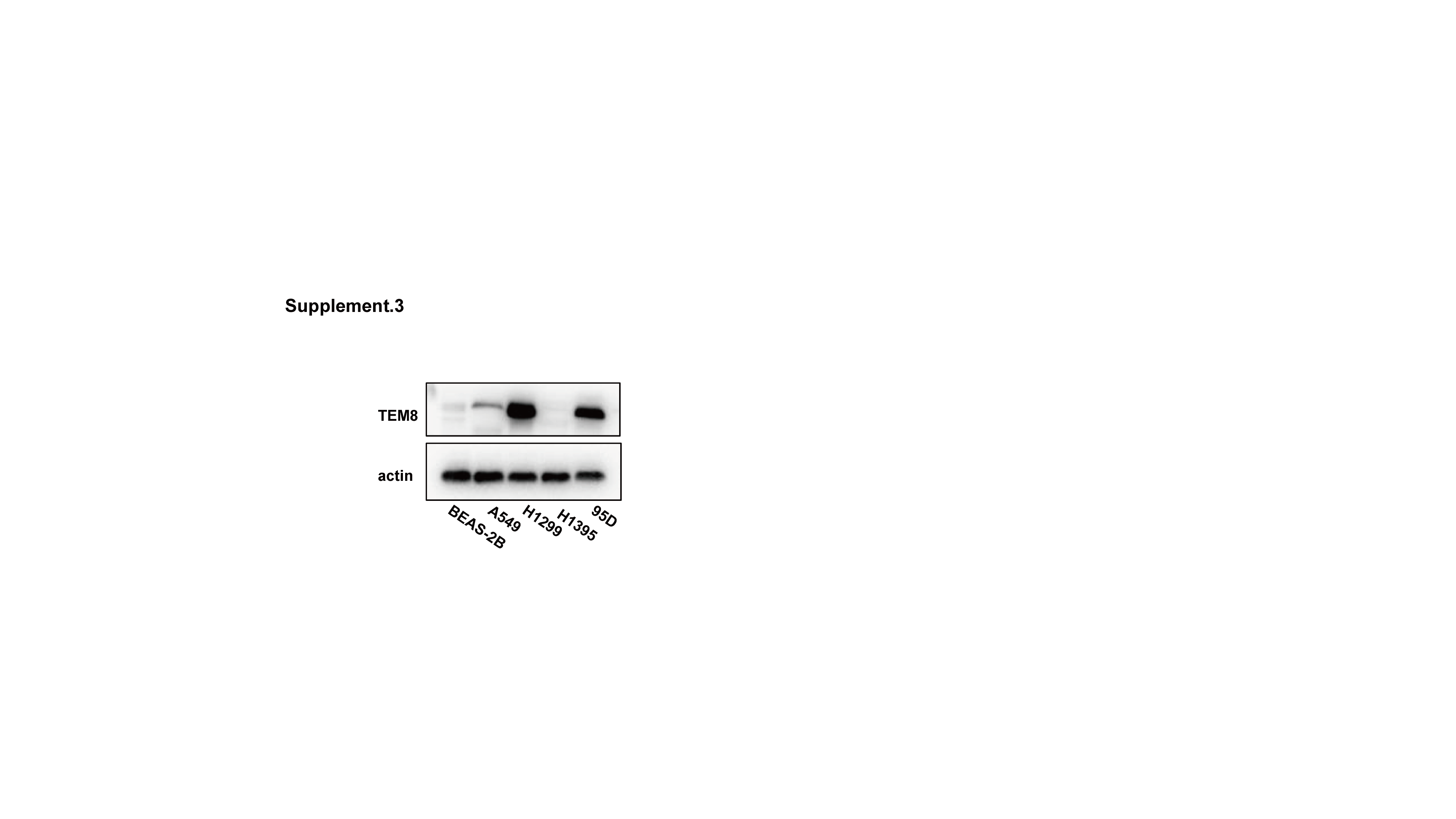

Supplement: Supplementary Figure 3 — The expression of TEM8 in lung cancer cell lines (A549, H1299, H1395, and 95D) and normal bronchial epithelial cell lines. [file Image_3.tif]
